# The effects of tennis on depressive symptoms and pro-social behaviors in university students: the mediating role of appreciative social support

**DOI:** 10.3389/fpsyg.2025.1428977

**Published:** 2025-03-20

**Authors:** Runjuan Sun, Tianpei Li, Mingyuan Li, Liang Meng

**Affiliations:** ^1^Department of Physical Education, Jeonbuk National University, Jeonju, Republic of Korea; ^2^Department of Sports Science, Jeonbuk National University, Jeonju, Republic of Korea; ^3^Suzhou University of Science and Technology, Suzhou, Jiangsu, China

**Keywords:** tennis, university student, depressive symptom, pro-social behavior, appreciating social support

## Abstract

**Aims:**

This research aimed to explore the impact of tennis on depressive symptoms and pro-social behaviors among college students, while also delving into the intermediary function of social support navigation.

**Materials and methods:**

Utilizing a suite of psychological evaluations and social support instruments, the study compared the levels of depressive symptoms and pro-social behaviors between collegiate tennis athletes and their non-athlete peers.

**Results:**

The findings revealed an inverse relationship between the duration of tennis engagement and the presence of depressive symptoms (*β* = −0.234, *p* <0.001), alongside a direct positive association with pro-social tendencies (*β* = 0.222, *p* < 0.001). Further scrutiny uncovered a substantial link between the degree to which participants valued social support and their experiences of depressive symptoms (*indirect effect* = −0.212, *95%CI* = −0.036 ~ −0.009) and pro-social behaviors (*indirect effect* = 0.025, *95%CI* = 0.009 ~ 0.044). Notably, the valuation of social support served as a conduit for the beneficial effects of tennis on these outcomes within the collegiate population. Consequently, the evidence from this investigation underscores the salutary influence of tennis on the psychological well-being and social conduct of college students, highlighting the pivotal role of understanding and leveraging social support.

**Conclusion:**

These insights offer valuable direction for fostering mental health and social proficiency in the university setting and advocate for the integration of sports as a viable component in mental health strategies.

## 1 Introduction

Over recent decades, depression has emerged as a significant mental health concern, drawing considerable scholarly interest worldwide. This issue is particularly acute within the college demographic, where the incidence of depression raises alarm (Husky et al., 2023). College students, navigating an era marked by an explosion of knowledge and rapid social transformation, encounter a myriad of stressors, including academic demands, career uncertainties, and complex social dynamics, all of which can precipitate depressive symptoms (Spatafora et al., 2022; Lei et al., 2021). Consequently, identifying effective strategies to mitigate these symptoms and bolster the mental well-being of university students has become a critical area of inquiry within mental health research.

Physical activity is universally acknowledged as a potent tool for enhancing mental health and diminishing the effects of depression (Pascoe and Parker, 2019; Dauwan et al., 2021). Tennis with its blend of collective physical exertion, skillful challenge, and social engagement, merits consideration for its potential mental health benefits. Beyond the physical benefits, tennis fosters social interaction, a key element in supporting the mental health of college students (Wang et al., 2019; Yaneva et al., 2020).

Moreover, tennis may influence depressive symptoms indirectly by bolstering perceived social support. Recognizing social support—the awareness of care and assistance from others—stands as a crucial buffer against mental health challenges (Khoury et al., 2021; Qi et al., 2020). Within the tennis milieu, players not only engage in physical activity but also experience camaraderie and encouragement, thereby enhancing their sense of social support. Social support has been highlighted as a key factor affecting mental health outcomes (Diotaiuti et al., 2021).

Additionally, fostering pro-social behaviors such as helping, sharing, and cooperating is vital among college students. These behaviors are instrumental in building positive social connections and are essential for successful social integration (Wang et al., 2021). Team-oriented sports like tennis create an optimal setting for nurturing these pro-social tendencies. Despite this, research exploring the influence of tennis on depressive symptoms and pro-social behaviors through the lens of perceived social support remains scarce. This study seeks to bridge this gap by investigating how tennis participation can alleviate depressive symptoms and encourage pro-social behaviors in college students by augmenting their perception of social support.

## 2 Materials and methods

### 2.1 Objects of study

A total of 150 general college students who play tennis (M = 20.62, SD = 1.67), and 150 professional tennis players (M = 20.65, SD = 1.72) participated in this study. The specific demographic information is shown in Table 1. The data were collected from 22 February to 3 March 2023 Participants in this study were completely voluntary and were not compensated. The study questionnaire was distributed electronically to participants through APP Survey Star (Changsha Ranxing Technology, China). The study was approved by the ethics committee of the first author’s institution. Participants were recruited through university-wide email invitations and posters displayed on campus. Data collection was conducted over a two-week period, during which participants completed an online survey hosted on Wenjuanxing. To ensure data quality, participants were required to provide informed consent before proceeding and were instructed to complete the survey in a single sitting. Measures were administered in a randomized order to minimize response bias. This study was approved by the Suzhou University of Science and Technology of Ethics Committee at Suzhou University of Science and Technology, with the approval number UTS2024001. All participants provided informed consent prior to participation, and the study adhered to the ethical guidelines outlined in the Declaration of Helsinki.

**Table 1:** Correlation of variables and descriptive statistical results.

	AB	PSS	DS	PSB
AB	1			
PSS	0.278**	1		
DS	−0.234**	−0.405**	1	
PSB	0.222**	0.437**	−0.138*	1

AB, Age of the Ball; PSS, Perceived Social Support; DS, Depressive Symptoms; PSB, Pro-Social Behavior. *p < 0.05; **p < 0.01.

### 2.2 Research tools

#### 2.2.1 Appreciating social support

The Perceived Social Support Scale (PSSS) revised by Jiang (1999) was used. The scale contains 12 entries and is scored on a 7-point scale, with higher scores indicating a higher level of social support felt by the individual. The Cronbach’s alpha coefficient in this study was 0.92.

#### 2.2.2 Depressive symptoms

The revised Centre for Epidemiological Studies Depression Scale (CES-D) by Chen et al. (2009) was used, which consists of 20 items and is scored on a 4-point scale, with higher total scores indicating a higher degree of depressive symptoms in an individual. The Cronbach’s alpha coefficient in this study was 0.89.

#### 2.2.3 Pro-social behavior

The Prosocial Tendencies Measure (PTM) was developed by Carlo and Randall (2002). The scale consists of 23 items with six dimensions and is assessed on a five-point scale, with higher scores indicating stronger tendencies for pro-social behavior. Kou et al. (2007) revised the Chinese version of the scale, which still retained the six dimensions of openness, anonymity, altruism, adherence, emotionality, and urgency, with a total of 26 items, and a Cronbach’s alpha coefficient of 0.88. The PTM was used as a proxy for the PTM.

### 2.3 Statistical analysis

All statistical analyses were conducted using SPSS 25.0 and PROCESS macro 3.4. Gender and socioeconomic status were included as control variables in all models to account for their potential confounding effects. Bootstrapping with 5,000 resamples was applied to test the significance of indirect effects in the mediation analysis, as well as to calculate confidence intervals for correlation and t-test results. Key coefficients obtained from the analyses were interpreted qualitatively to provide insights into their practical implications. For example, a standardized regression coefficient of 0.35 indicates a moderate positive relationship between appreciative social support and prosocial behavior.

## 3 Results

### 3.1 Correlation and descriptive statistics of variables

Table 1 presents the results of the correlation analysis and descriptive statistics between the variables, as shown by the significant positive correlation between the age of the ball and perceived social support, the significant negative correlation between the age of the ball and depressive symptoms, and the significant positive correlation between the age of the ball and pro-social behavior. There was a significant negative correlation between navigational social support and depressive symptoms and a significant positive correlation between navigational social support and pro-social behavior. There was a significant negative correlation between depressive symptoms and pro-social behavior.

### 3.2 Analysis of differences between professional and non-professional players on each variable

Table 2 presents the differences between ordinary college students and professional tennis players, and it was found that the age of tennis players was significantly higher than that of ordinary college students (*t* = −2.380, *p* = 0.018), the level of perceived social support was significantly higher than that of ordinary college students (*t* = −2.340, *p* = 0.020), and the level of pro-social behaviors of tennis players was significantly higher than that of ordinary college students (*t* = −2.570, *p* = 0.011). There was no significant difference between the levels of depressive symptoms of ordinary university students and tennis players.

**Table 2:** Difference analysis table.

	Rank	N	Mean	SE	*t*	*p*	Cohen’s *d*
AB	Non-tennis player	150	3.41	2.03	−2.38	0.018	−0.275
	Tennis player	149	3.97	2.04			
PSS	Non-tennis player	150	3.94	0.71	−2.34	0.020	−0.269
	Tennis player	149	4.13	0.70			
DS	Non-tennis player	150	2.55	0.46	0.501	0.617	0.070
	Tennis player	149	2.52	0.39			
PSB	Non-tennis player	150	2.93	0.46	−2.57	0.011	−0.292
	Tennis player	149	3.06	0.43			

### 3.3 Analysis of the role of intermediaries

#### 3.3.1 Appreciation of the mediating role of social support in the relationship between ballistic age and depressive symptoms

**Figure 1:**
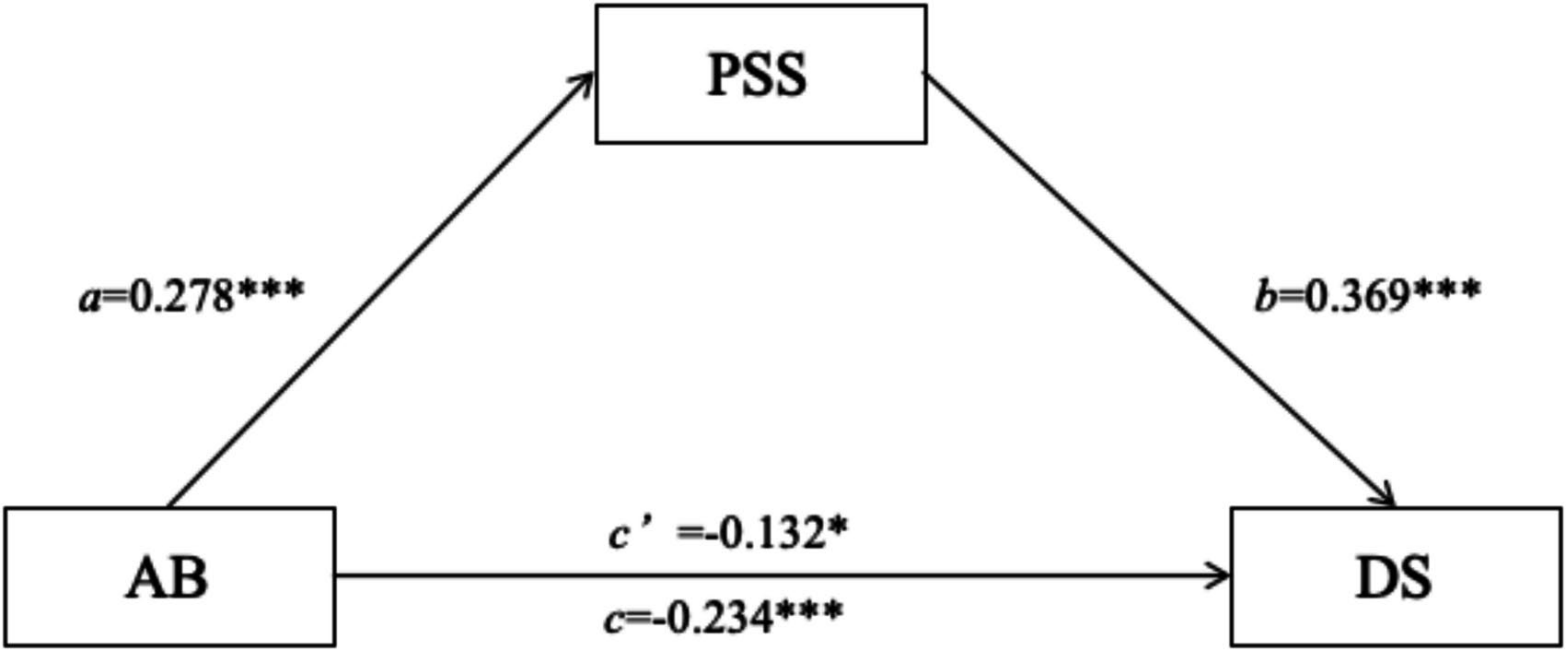
The mediating role of social support between playing age and depressive symptoms. *p < 0.05, ***p < 0.001.

Age had a significant positive effect on perceptual social support (*β* = 0.278, *p* < 0.001), i.e., the longer the time spent playing tennis, the higher the level of perceptual social support. Age (*β* = −0.132, *p* = 0.017) and perceived social support (*β* = −0.369, *p* < 0.001) had a significant negative effect on depressive symptoms, i.e., the longer the time spent playing tennis, the lower the level of depression, and the higher the level of perceived social support, the lower the level of depression. Thus, it can be shown that comprehending social support partially mediates the relationship between ballistic age and depression (Figure 1; Tables 3, 4).

**Table 3:** The mediating role of social support between playing age and depressive symptom.

Variable	DS			PSSS			DS		
	*β*	95%CI	*p*	*β*	95%CI	*p*	*β*	95%CI	*p*
AB	−0.234	−0.345 ~ −0.123	<0.001	0.278	0.168 ~ 0.388	<0.001	−0.132	−0.239 ~ −0.024	0.017
PSS							−0.369	−0.477 ~ −0.261	<0.001
R	0.234			0.278			0.425		
R2	0.055			0.077			0.18		
F	17.208***			24.868***			32.544***		

Mediating effect (a*b) = −0.102. ***p < 0.001.

**Table 4:** Bootstrap.

	Effect	BootSE	LLCI	ULCI	Percent
Total	−0.234	0.056	−0.345	−0.123	100%
Direct effect	−0.132	0.055	−0.239	−0.024	56.4%
AB→PSS → DS	−0.102	0.036	−0.178	−0.039	43.6%

#### 3.3.2 The mediating role of appreciative social support in the relationship between ballroom age and pro-social behavior

**Figure 2:**
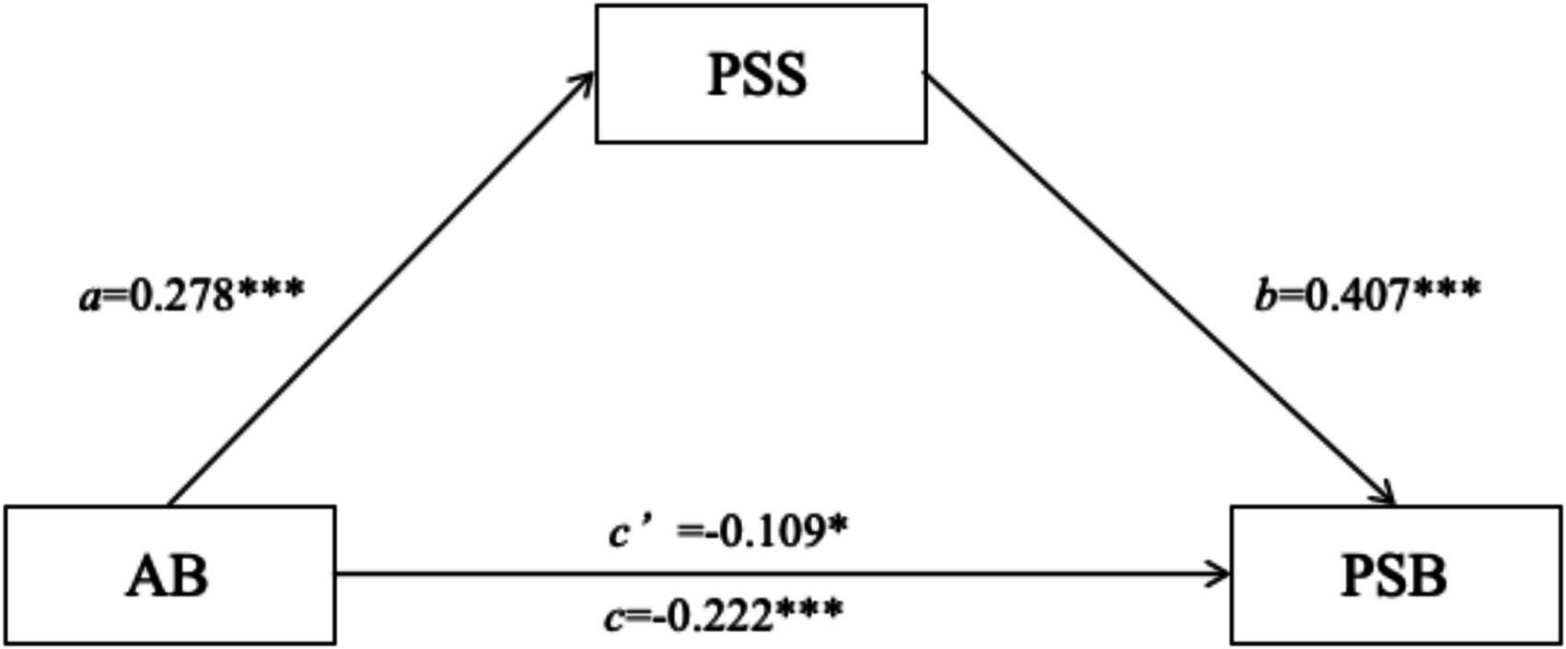
The mediating role of social support between playing age and prosocial behavior. *p < 0.05, ***p < 0.001.

Age had a significant positive effect on perceptual social support (*β* = 0.278, *p* < 0.001), i.e., the longer the time spent playing tennis, the higher the level of perceptual social support. Age (*β* = 0.109, *p* = 0.045) and perceived social support (*β* = 0.407, *p* < 0.001) had a significant positive effect on pro-social behavior, i.e., the longer the time spent playing tennis, the higher the pro-social behavior, and the higher the level of perceived social support, the higher the pro-social behavior. It can therefore be shown that comprehending social support partially mediates the relationship between ball age and pro-social behavior (Figure 2; Tables 5, 6).

**Table 5:** The mediating role of social support between playing age and prosocial.

Variable	PSB			PSS			PSB		
	*β*	95%CI	*p*	*β*	95%CI	*p*	*β*	95%CI	*p*
AB	0.222	0.111 ~ 0.333	<0.001	0.278	0.168 ~ 0.388	<0.001	0.109	0.002 ~ 0.215	0.045
PSS							0.407	0.301 ~ 0.513	<0.001
R	0.222			0.278			0.45		
R2	0.049			0.077			0.202		
F	15.384***			24.868***			37.484***		

Mediating effect (a*b) = 0.113. ***p < 0.001.

**Table 6:** Bootstrap.


	Effect	BootSE	LLCI	ULCI	Percent
Total	0.222	0.057	0.111	0.333	100%
Direct effect	0.109	0.054	0.002	0.215	49.1%
AB→PSS → PSB	0.113	0.042	0.042	0.209	50.9%

## 4 Discussion

The results of this study suggest that playing age can significantly reduce depressive symptoms and also significantly increase prosocial behaviors in athletes. It is understood that social support plays a mediating role in it.

### 4.1 Influence of age of ball on depressive symptoms in tennis players

In evaluating the influence of tennis-playing duration on depressive symptoms among university students, our study found a noticeable correlation. The longer the duration that students had been playing tennis, the lesser were their depressive symptoms. This relationship between physical activity and depression has been well-documented in literature, reinforcing our findings (Craft and Perna, 2004). The relationship can be partially explained by the biological theory which suggests that physical activities like tennis lead to the release of endorphins, also known as “feel good” hormones, in the brain (Dinas et al., 2011). Regular release of these endorphins through sustained involvement in tennis could therefore potentially decrease depressive symptoms over time. Moreover, the psychological theory posits that physical activities provide a distraction from worries and negative thoughts that feed depression (Scully et al., 1998). Given the mental concentration required in tennis, it is plausible that longer duration of play provides a greater respite from such negative cognitions, thus reducing depressive symptoms. Furthermore, the social interaction theory suggests that sports like tennis, which require interpersonal communication and cooperation, provide an opportunity for social interaction, helping to combat feelings of loneliness and isolation that are often associated with depression (Paluska and Schwenk, 2000). Long-term tennis players are likely to have established a solid social network within the sport, which may provide them with strong social support, thereby acting as a buffer against depression. However, it is important to note that while our study and the aforementioned literature suggest a protective effect of long-term tennis playing against depressive symptoms, it does not necessarily imply causality. Other factors, such as individual personality traits and external support systems, might also play significant roles in this context (Nimrod and Kleiber, 2007).

In conclusion, our findings suggest that the longer duration of tennis playing could potentially reduce depressive symptoms in university students, possibly through the combined effects of biological, psychological, and social factors. These findings underscore the importance of promoting regular and long-term participation in sports like tennis for the mental wellbeing of university students.

### 4.2 Influence of age of ball on pro-social behaviors in tennis players

In our study, we observed that the duration of tennis participation positively correlated with pro-social behaviors among university students. This suggests that the longer a student had been involved in playing tennis, the more likely they were to demonstrate pro-social behaviors such as cooperation, sharing, and helping others. This finding is consistent with prior research suggesting that involvement in team sports promotes pro-social behavior (Eime et al., 2013). One potential explanation for this relationship is the social nature of tennis. Tennis often involves doubles play, where cooperation and communication with a partner are essential. These repeated interactions over time can foster a sense of teamwork and mutual understanding, which are key components of pro-social behavior (Bruner et al., 2014). Further, longer-term involvement in tennis may also provide more opportunities for athletes to develop and refine their social skills. Social interaction is an inherent part of tennis, and over time, players may become more adept at negotiating, compromising, and empathizing with others - all of which are important aspects of pro-social behavior (Kavussanu and Boardley, 2009). However, it is worth noting that while our findings suggest a positive relationship between tennis-playing duration and pro-social behaviors, it does not imply causality. Other factors such as individual personality traits, coaching styles, and team dynamics could also influence the development of pro-social behaviors in athletes (Hodge and Lonsdale, 2011).

In conclusion, our study adds to the body of evidence suggesting that long-term participation in sports like tennis may enhance pro-social behaviors in university students. However, further research is needed to fully understand the underlying mechanisms of this relationship.

### 4.3 The mediating role of perceive social support

Our study also sought to understand the mediating role of appreciative social support in the relationship between tennis-playing duration and both depressive symptoms and pro-social behaviors. The results demonstrated that appreciative social support significantly mediated these relationships, a finding consistent with previous research (Rees and Hardy, 2000). Appreciative social support, derived from the tennis environment, could be a critical factor in reducing depressive symptoms. The social interactions and positive reinforcement received from coaches, teammates, and even spectators might foster a sense of belonging and acceptance (Sarason et al., 1997). This feeling could counteract feelings of isolation and loneliness, common triggers for depressive symptoms. Over time, continued involvement in tennis and the associated social support could potentially lead to a decrease in depressive symptoms (Cohen and Wills, 1985). Similarly, appreciative social support could play a significant role in enhancing pro-social behaviors among tennis players. The social nature of tennis often requires cooperation and mutual understanding, and positive social support could encourage these behaviors. Players who feel appreciated and supported are likely to reciprocate these positive behaviors toward others, promoting a cycle of pro-social behavior within the tennis community (Bandura, 1977). However, it is important to consider other factors such as personality traits and socio-economic status which can also influence the perception and effect of social support (Lakey and Cohen, 2000). More research is needed to fully understand the complexity of these relationships.

In conclusion, our findings highlight the potential mediating role of appreciative social support in the relationship between tennis-playing duration, depressive symptoms, and pro-social behaviors. It suggests that fostering a supportive environment in sports settings could be beneficial for athletes’ mental health and social interactions.

### 4.4 Limitation

This study has several limitations. First, the sample was limited to college students in a specific region, possibly limiting the generality of the results. Second, the data relies largely on self-reporting, which can lead to social expectation effects or memory biases. The cross-sectional design of the study did not allow causal relationships to be established, only correlations between variables were revealed. In addition, only the effects of tennis were examined, and the potential effects of other sports types were not considered. Understanding social support as a mediating variable may be influenced by unmeasured factors, such as personality traits or socioeconomic status. Finally, without considering the effects of long-term participation in tennis, short-term studies may not reflect long-term effects, and insufficient consideration of the impact of cultural context on social support and mental health may limit the applicability of the results in different cultural contexts.

## 5 Conclusion

The aim of this study was to investigate the effects of tennis on depressive symptoms and pro-social behavior in university students and to examine the mediating role of comprehending social support. By collecting relevant psychological assessments and social support questionnaires from the participants, we reached the following conclusions: (1) Tennis has a significant mitigating effect on depressive symptoms in college students. The results of the study showed that the longer the time spent playing tennis, the less depressive symptoms. This suggests that tennis, as a physical activity, has a positive impact on improving the mental health of college students. (2) Tennis has a positive impact on the pro-social behavior of university students. The results of the study showed that the longer the time spent playing tennis, the higher the level of pro-social behavior. This suggests that by participating in tennis, university students are more inclined to be socially active, caring and supportive. (3) Appreciation of social support mediated the effects of tennis on college students’ depressive symptoms and pro-social behavior. Through further analyses, we found significant correlations between participants’ perceptions of social support and depressive symptoms and pro-social behavior during tennis training. This suggests that participants’ level of appreciation of social support is an important factor in the impact of tennis on their mental health and pro-social behavior.

In summary, the results of this study indicate that tennis has a significant mitigating effect on college students’ depressive symptoms and promotes the development of pro-social behaviors. In addition, comprehending social support plays a mediating role in this process. These findings provide theoretical and practical guidance for the mental health and social development of college students, as well as empirical support for the application of sport in the field of mental health. Provide more actionable recommendations for college administrators and mental health professionals to suggest specific strategies for integrating tennis or similar activities into wellness programs. Summarize how this study extends existing literature to highlight the practical importance of creating a supportive environment in sport.

## Data Availability

The original contributions presented in the study are included in the article/supplementary material, further inquiries can be directed to the corresponding author.
